# Characterizing Phage-Host Interactions in a Simplified Human Intestinal Barrier Model

**DOI:** 10.3390/microorganisms8091374

**Published:** 2020-09-07

**Authors:** María A. Núñez-Sánchez, Joan Colom, Lauren Walsh, Colin Buttimer, Andrei Sorin Bolocan, Rory Pang, Cormac G. M. Gahan, Colin Hill

**Affiliations:** 1 APC Microbiome Ireland, Bioscience institute, University College Cork, T12 YT20 Cork, Ireland; mariaangeles.nunezsanchez@ucc.ie (M.A.N.-S.); joan.colomcomas@ucc.ie (J.C.); 114434442@umail.ucc.ie (L.W.); colin.buttimer@ucc.ie (C.B.); andrei.s.bolocan@gmail.com (A.S.B.); rorypang@ucc.ie (R.P.); C.Gahan@ucc.ie (C.G.M.G.); 2 School of Microbiology, University College Cork, T12 YN60 Cork, Ireland; 3 School of Pharmacy, University College Cork, T12 YN60 Cork, Ireland

**Keywords:** *Enterococcus faecalis*, bacteriophage, *Herelleviridae*, intestinal model, phage therapy, IBD

## Abstract

An intestinal epithelium model able to produce mucus was developed to provide an environment suitable for testing the therapeutic activity of gut bacteriophages. We show that *Enterococcus faecalis* adheres more effectively in the presence of mucus, can invade the intestinal epithelia and is able to translocate after damaging tight junctions. Furthermore, *Enterococcus* phage vB_EfaM_A2 (a member of *Herelleviridae* that possesses virion associated immunoglobin domains) was found to translocate through the epithelium in the presence and absence of its host bacteria. Phage A2 protected eukaryotic cells by reducing mortality and maintaining the structure of the cell layer structure. We suggest the mammalian cell model utilized within this study as an adaptable in vitro model that can be employed to enable a better understanding of phage–bacteria interactions and the protective impact of phage therapy relating to the intestinal epithelium.

## 1. Introduction

Inflammatory bowel disease (IBD) includes both ulcerative colitis (UC) and Crohn’s disease (CD) and affects an estimated two million people in Europe [1]. It is a chronic inflammatory disorder with no single known cause, although it is has been suggested to result from intestinal bacteria inducing an unwarranted activation of the gut’s mucosal immune system [2]. This aberrant response may be as a result of a reduction in commensal bacteria and an increase in potentially pathogenic bacteria [3]. Most of the treatments developed for IBD use small molecules, vaccines, anti-TNF, or monoclonal antibodies to modulate the immune response in the gut [4,5,6]. Unfortunately, these therapies fail for a third of patients, most likely because of the complexity of the disease and the suggested imbalance of the gut microbiota [7,8,9]. Several groups of bacteria have been implicated and there is a correlation between increased *Enterococcus faecalis* levels in patients with IBD, and in particular in those with Crohn’s disease [10]. These *E. faecalis* strains have been reported to share genes involved in protease gelatinase activity, which play a crucial role in pathological alterations following its infection [11,12]. Gastrointestinal colonization with strains having this activity could potentially impact the tight junctions between epithelial cells and subsequently disrupt the mucosal barrier and trigger inflammation in the gut [13]. Targetting and reducing levels of such bacterial strains like *E. faecalis* could potentially help to alleviate IBD symptoms.

Bacteriophage (phage) therapy has shown to be an effective treatment for *E. faecalis* infections and has been used to treat human prostatitis linked to *E. faecalis* in three different patients, which resulted in complete eradication of *E. faecalis* in all three subjects [14]. Phages have also been successfully used to treat *E. faecalis* induced sepsis [15] and *Enterococcus faecium* bacteraemia [16]. Moreover, this type of therapy was able to attenuate alcoholic liver disease promoted by cytolytic *E. faecalis* found in the intestinal tract [17]. Another point worth noting is the general known to be lower, or absence of collateral damage caused by phages on the residual or commensal microbiota in comparison of antibiotics as antibacterial agents [18,19]. However, there does exist a discussion if whether certain types of phages infecting particular bacteria species could be involved in certain human pathologies [20,21].

With the increasing need for new therapeutics globally and especially in the light of the worldwide increasing bacterial antibiotic resistances, phages as treatment agents should be studied further in-depth as compliments or alternatives. However, candidate phages and host pathogens are usually characterized under planktonic conditions in a laboratory environment. Under such conditions, it is difficult to predict their behavior in contact with the epithelial cell barrier and in the presence of a mucosal layer in the intestinal tract. These features could significantly affect the interaction between the host, the bacterium, and the phage with consequences for the intestinal epithelium and the integrity of the trans-epithelial barrier.

Not all phages can be deemed suitable for therapeutic applications. It is generally regarded that such phage should be virulent, produce a high yield of phage per infected cell, be specific for the problematic bacteria of interest but yet possess broad host range to infect numerous strains, have good shelf life without loss of lytic activity, as well not possessing genes that encode for virulence factors [22]. Phages from the family *Herelleviridae* how been found to possess such characteristics and are being used in phage preparations for clinical applications. However, limited data exists regarding their interaction with mucosal surfaces [23,24].

We set out to develop an in vitro model reproducing features of the gastrointestinal epithelium and the mucus layer in an attempt to characterize the interaction between phage, a pathogenic intestinal bacteria and eukaryotic cells in the gut. Considering the relevance of *E. faecalis* in IBD, its impact over tight junctions and the availability of suitable phage, this bacterium was selected to validate the model. A co-culture of Caco-2 and HT29-MTX-E12 (HT29-MTX) cells was used to mimic the epithelium and mucus layer. This layer was infected with an *E. faecalis* and treated with phage A2, before testing the levels of cell viability, *E. faecalis,* and phage adherence and transcytosis.

## 2. Materials and Methods

### 2.1. Bacterial Strains and Phage and Cultivation Techniques

Phage A2 was initially isolated from farm animal faeces by an enrichment method. Animal faecal matter was placed into tryptic soy broth (TSB) with an overnight culture of *E. faecalis* OG1RF and then incubated overnight at 37 °C with shaking. This was then centrifuged to pellet faecal matter with supernatant then being filtered (0.45 µm pore-size filter, Sarstedt, Nümbrecht, Germany). The supernatant was spotted onto TSB overlay seeded with *E. faecalis* strain OG1RF. The phage was isolated by picking an individual plaque followed by replating and reisolating to ensure purity [25]. *Enterococcus* phage A2 was routinely cultivated at 37 °C by the soft agar overlay method [26] using TSB (Merck KGaA, Darmstadt, Germany) agar plates (1% *w*/*v*) and TSB overlays (0.2% *w*/*v* agarose, Sigma-Aldrich, Wicklow, Ireland) supplemented with 10 mM MgSO4. *E. faecalis* strain OG1RF was used as the host to propagate the phage (ATCC culture collection). *E. faecalis* strain OG1RF was routinely cultured in TSB at 37 °C. Bacterial counts were obtained by serial diluting of the strain in phosphate saline buffer (PBS), plating onto TSB agar (1%), and incubating at 37 °C overnight. For the enumeration of the phage A2, salt magnesium (SM) buffer was used for serial dilutions. Twenty microliters of the appropriate phage dilution were added to 100 μL of an overnight culture of *E. faecalis* OG1RF. This mixture was then inoculated to 3 mL of TSB overlay (0.2% agarose) containing calcium boro-gluconate and magnesium sulphate at a final concentration of 10 mM. Finally, the mixture was poured onto TSB agar plates (1%) and incubated at 37 °C overnight before counting the phage. Alternatively, 100 μL of *E. faecalis* OG1RF was added to 3 mL of the supplemented TSB overlay (0.2% agarose), poured onto TSB agar plates (1%), and let set. Ten microliters of the phage lysate were then spotted onto the agar and let dry before incubating overnight at 37 °C.

### 2.2. Phage Propagation

Ten milliliters of TSB broth were inoculated with 100 μL of an overnight culture of *E. faecalis* OG1RF. Following 3 h of incubation shaking at 150 rpm at 37 °C, the phage was added at a final multiplicity of infection (MOI) of 1. The culture was incubated for a further 3–4 h at 37 °C or until lysis before centrifuging it at 5000× *g*. The supernatant was then filter sterilized through a 0.45 μm syringe filter (Sarstedt, Nümbrecht, Germany). The lysate was then titrated and stored at 4 °C.

### 2.3. Cscl Gradient Purification and Transmission Electron Microscopy

Phage lysate underwent centrifugation at 107,328× *g* for at 4 °C for 2 h using an F65L-6x13.5 rotor (ThermoScientific, Waltham, Massachusetts, United States). The resulting phage pellets were resuspended in SM buffer (50mM Tris-HCl pH 7.5, 100mM NaCl, 8 mM MgSO_4_ 7H2O). This phage preparation was then placed onto a CsCl step gradient composed of 1.3, 1.4, and 1.5 g/mL layers and spun at 107,328× *g* for 4 °C for 2.5 h using an F65L-6x13.5 rotor. Resulting bands were collected and desalted using Amicon Ultra-0.5 Centrifugal Filter Units with a 3 kDa Molecular weight cut-off (MWCO) membrane (Merck, Cork, Ireland). Following this, the purified phage preparation was placed onto a Formvar/Carbon 200 Mesh, Cu grids (Electron Microscopy Sciences) with subsequent removal of the excess sample by blotting. Grids were then negatively contrasted with 0.5% (*w*/*v*) uranyl acetate and examined at UCD Conway Imaging Core Facility (University College Dublin, Dublin, Ireland) by a transmission electron microscope.

### 2.4. DNA Extraction, Library Preparation, and Sequencing

A phage lysate was precipitated using polyethylene glycol (15% *w/v* PEG8000, 1 M NaCl) at 4 °C overnight and centrifuged, after which the pellet was resuspended in SM buffer. The resulting phage particles were then treated with DNase I (ThermoFisher Scientific, Waltham, MA, USA) and RNase I (ThermoFisher Scientific, Waltham, MA, USA) prior to genome DNA extraction using a commercial phage DNA isolation kit (Norgen, Thorold, ON, Canada) and subsequent clean up using a commercial DNA clean up kit (Promega, Madison, WI, USA), used following the manufacturer’s protocols. DNA was then quantified using the Qubit broad range assay (ThermoFisher Scientific, Waltham, MA, USA) before standardization for paired-end Nextera XT library preparation (Illumina, San Diego, CA, USA). Library quality was inspected with Agilent Technology 2100 Bioanalyzer a High Sensitivity DNA chip before sequencing with an Illumina MiSeq platform (Illumina Inc., San Diego, CA, USA). Genome de novo assembly was performed using default parameters with MetaSPAdes (St. Petersburg, Russia).

### 2.5. Bioinformatic Analysis

The RAST server (http://rast.nmpdr.org/; [27]) was employed with GLIMMER [28] to predict open reading frames (ORFs). With further analysis of ORFs conducted with BLASTP (http://blast.ncbi.nlm.nih.gov/Blast.cgi?PAGE=Proteins), Pfam (http://pfam.xfam.org/search#tabview=tab1; [29]), Interproscan [30]; and HHpred (https://toolkit.tuebingen.mpg.de/#/tools/hhpred; [31]). With the detection of ORFs with transmembrane domains, signal peptide sequence and lipoprotein cleavage signal being identified with the use of TMHMM v.2 (http://www.cbs.dtu.dk/services/TMHMM/; [32]), SignalP v.4.1 (http://www.cbs.dtu.dk/services/SignalP/; [33]), and LipoP v.1 (http://www.cbs.dtu.dk/services/LipoP/; [34]), respectively. Translated ORFs from phage A2 were searched against hidden Markov model profiles downloaded from the Prokaryotic Virus Orthologous Groups (pVOGs) database [35] using hmmscan (v.3.1b2) [36] with an E-value cut off of 1 × 10^−5^. Screening for antibiotic resistant genes was achieved with ABRicate (https://usegalaxy.eu/) and PHACTS to predict phage lifestyle (http://edwards.sdsu.edu/PHACTS/index.php; [37]. The Artemis Comparison Tool (v.16.0.0) [38] was used for the identification of shared structural proteins of *Enterococcus* phage A2 to *Staphylococcus* phage ISP (accession no. FR852584) and *Listeria* phage A511 (accession no. NC_009811) with confirmation of homology using BLASTP. The molecular weights of the predicted ORFs were estimated using the batch protein molecular weight determination of the sequence manipulation suite (http://www.bioinformatics.org/sms2/protein_mw.html). The presence of transfer RNA genes was investigated with the use of ARAGORN (http://130.235.46.10/ARAGORN/; [39]). The circular genome map of the phage was drawn using GCview [40].

### 2.6. Phylogenetic and Comparative Analysis

Genome comparison between phages was visualized with the use of Easyfig [41] with comparisons between genomes being made with TBLASTX. A total proteome comparison between phages was conducted using Coregenes 3.5 with the BLASTP threshold set at 75% http://binf.gmu.edu:8080/CoreGenes3.5/; [42]). Heat map comparing the genomes was generated using Gegenees [43], using TBLASTX or BLASTN with accurate parameters (fragment length: 200 bp; step size: 100 bp; threshold: 0%). VICTOR was employed for all pairwise comparisons between phages at the amino acid level which employs the Genome-BLAST Distance Phylogeny (GBDP) method [44] under settings recommended for prokaryotic viruses [45]. The resulting intergenomic distances (including 100 replicates each) were used to infer a balanced minimum evolution tree with branch support via FASTME, including SPR postprocessing [46] for each of the formulas D0, D4, and D6, respectively. The phylogenetic trees (with branch support) were rooted at the midpoint and visualized with iTOL [47]. Ig-like domains were determined by reannotation of all phage genomes with Prokka (v.1.11) [48]. Phage proteins were then compared to the Pfam database using hmmscan with an E-value cut off of 1 × 10^−5^.

### 2.7. Phage A2 Infectivity in Cell Culture Media

Ten milliliters of TSB broth was inoculated with 100 μL of an overnight culture of *E. faecalis* OG1RF. The solution was mixed and incubated 37 °C with shaking (150 rpm). At the same time, a second culture was infected with the phage A2 after three hours of incubation. The multiplicity of infection (MOI) was adjusted to 1. Samples were taken every hour for 7 h to check optical density (OD), bacterial and phage counts. A 100 μL sample was taken and added to 900 μL of TSB in a cuvette to take OD_600nm_ measurements. Bacterial and phage counts were carried out as previously described. The same experiment was also carried out using the cell culture media Dulbecco’s Modified Eagle Medium (DMEM) with Glutamax supplemented with 15% fetal bovine serum (FBS) and 1% non-essential amino acids.

### 2.8. Cell Lines, Culture Conditions, and Treatments

The mucus-producing HT29-MTX-E12 (HT29-MTX) cell line was obtained from the European Collection of Authenticated Cell Cultures (ECACC, 12040401). The enterocyte-like Caco-2 cells were obtained from the American Type Culture Collection (ATCC, Rockville, MD, USA). Cell lines were grown in Dulbecco’s Modified Eagle’s medium (DMEM; 4.5 g/L D-glucose) supplemented with 10% v/v FBS, 100 U/mL penicillin and 100 µg/mL streptomycin, and 1% MEM non-essential amino acids (Sigma-Aldrich, Wicklow, Ireland). Cells were maintained at 37 °C in an incubator under a 5% CO2 and 95% relative humidity. Cells were sub-cultured 1–2 times a week with Trypsin-EDTA (Sigma-Aldrich, Wicklow, Ireland), counted using a hemocytometer and seeded at a density of approximately 15,000 cells cm^−2^.

For the assays, a co-culture of Caco-2 and HT29-MTX (4:1 ratio) cells were seeded at a density of 50,000 cells/cm^2^ for the short-time experiments (no mucus) and incubated for 24 h in growth media containing DMEM Glutamax (GIbco, Biosciences, Dublin, Ireland) supplemented with 15% *v/v* FBS, 100 U/mL penicillin, 100 µL/mL streptomycin, and 1% *v/v* non-essential amino acids. For the long-term experiments cells were seeded at a density of 15,000 cells/cm^2^ for the long-term experiments and incubated in growth media for 20 days. Under these conditions, mucus-producing Ht29-MTX cells are able to produce a continuous mucus layer [49]. Once the cultures were ready to treat, the medium was removed and cells were washed with Dulbecco’s phosphate buffer saline (DPBS) before adding fresh antibiotic-free medium containing 10^6^ CFU/mL *E. faecalis*. After 3 h incubation, the medium was removed and cells were washed three times with DPBS before the addition of new medium containing 10^6^ PFU/mL phage A2. Control cells and cells exposed to *E. faecalis* without the phage treatment were also run in parallel and subjected to the same changes in medium and washes (Supplementary information 1, Figure S1). Another control treated with phage A2 and without pre-treatment with *E. faecalis* was included.

### 2.9. E. faecalis Lethal Dose for Cell Lines and Cell Viability Assays

To select the appropriate dose of *E. faecalis* OG1RF to carry on the experiments, Caco-2 and HT29-MTX cells were separately seeded in 96 well-plates at a density of 50,000 cells cm^−2^ and grown for 24 h. Cells were then washed with DPBS and fresh medium containing *E. faecalis* at concentrations 10^9,^ 10^8^, 10^7^, 10^6^,10^5^, 10^4^, and 10^3^ CFU/mL was added to the wells. After the addition of the bacteria, plates were incubated for 4 h at 37 °C and then washed three times with DPBS. Following incubation, cell viability was determined using the CellTiter-Glo^®^ luminescent cell viability assay (Promega, MyBio, Ireland) according to the manufacturer’s conditions. Briefly, after the treatments, the wells were washed three times to remove the bacteria in the supernatant and 100 µL of fresh medium was added to each well. Plates were placed at room temperature for 30 min to equilibrate, then 100 µL of the CellTiter-Glo^®^ was added to each well. The contents were mixed for 5 min using an orbital shaker at 200 rpm. The plates were allowed to stabilize for 15 min at room temperature and luminescence was measured using a BioTek Synergy 2 microplate reader (BioTek, Winooski, VT, USA). Control wells contained cells without bacteria and subjected to the same procedures.

Cell viability was evaluated after the treatments using the same protocol above described. Untreated cells and cells treated only with *E. faecalis* were run in parallel and subjected to the same procedures in all the experiments. In addition, to evaluate if the phage had an impact on cell viability, wells containing phage but without the pre-treatment with *E. faecalis* were included in all the experiments.

### 2.10. Determining Trans-Epithelial Electrical Resistance (TEER)

Trans-epithelial electrical resistance (TEER) was measured for the evaluation of tight junction disruption during *E. faecalis* invasion. Caco-2/HT29-MTX co-cultures were seeded on 0.4 µm polyethylene terephthalate (PET) hanging inserts (Millicell, Merck) in 24-well plates (Corning) at a seeding concentration of 5000 cells cm^-2^ (80% Caco-2, 20% MTX-HT29). After 20 days of cultivation, cells were washed once with DPBS and 200 µL of DMEM Glutamax medium containing 15% *v*/*v* FBS and 1% *v*/*v* non-essential amino acids were added to the apical chamber. The basolateral compartments were filled with 1.3 mL of the same medium. TEER values were measured before treating the cells (*t* = 0 h). Twenty microliters of cell medium was removed from the apical chamber in the corresponding wells prior to the addition of 20 µL of DMEM Glutamax containing 10^6^ CFU/mL of *E. faecalis*. After 3 h incubation, the cells were washed once with DPBS, topped with fresh medium with or without phage A2, and incubated for another 21 h. After the treatments, the cell medium was removed, cells were washed once with DPBS, and fresh medium was added to both apical and basolateral chambers. The plates were incubated for 30 min to allow them to stabilize and then TEER values were determined. Changes in TEER were calculated as a percentage of initial TEER at time 0 h. Only wells with TEER values above 200 Ω·cm^2^ were included. TEER assays were run in triplicate and in three independent experiments.

### 2.11. Determining Adherence and Invasion of E. faecalis to Cells Following Phage Treatment

In order to investigate the adhesion and invasion capacity of *E. faecalis* following phage treatment, Caco-2/HT29-MTX co-cultures (80:20) were seeded in 96-well plates at a density of 50,000 cells/cm^2^ and incubated for 24 h in antibiotic-free DMEM Glutamax medium (15% FBS and 1% non-essential amino acids). Two hundred μL of DMEM Glutamax containing *E. faecalis* at a concentration of 10^6^ CFU/mL was added to each well. After a 3 h incubation at 37 °C, supernatants were removed and cells were washed three times with DPBS before adding 200 μL of fresh media containing the phage adjusted at a final MOI of 1. The mixture was incubated during 3 or 24 h at 37 °C. Before collecting the samples, the cells were washed three times with DPBS to remove any remaining bacteria in the supernatant. This was followed with incubation with 200 μL of DMEM Glutamax containing 75 μg/mL of Penicillin G (Sigma-Aldrich Aldrich, Wicklow, Ireland) for 2 h to kill extracellular *E. faecalis*. Cells were then disrupted with 0.1% Triton X-100 in DPBS, homogenized, and incubated at room temperature for 10 min. Then, a 1:4 dilution with TSB medium was done. Serial dilutions of the disrupted cell suspensions and the supernatants were plated on TSB agar to count the numbers of *E. faecalis* and phage A2. The plates were incubated overnight at 37 °C. To evaluate the number of adhered *E. faecalis*, the same protocol was used but without the penicillin G treatment. The same assays were performed with differentiated cells producing mucus. In this case, Caco-2/HT29-MTX co-cultures were plated in PET hanging inserts (Millicell, Merck, Kenilworth, NJ, USA) in 24-well plates at a density of 5000 cells/cm^−2^ and grown until the co-culture had reached full differentiation (20 days). Adhesion and invasion assays were performed as described above.

### 2.12. Accession Number

The genome sequence of *Enterococcus* phage A2 was submitted to GenBank under accession number MT856905.

### 2.13. Statistical Analysis

All statistical analysis was done using GraphPad Prism v.5. Normality of samples was determined using the Shapiro–Wilk test. All parametrically distributed samples were tested with the one-way ANOVA test followed by the post hoc Bonferroni test. For samples following a non-parametric distribution the Kruskal–Wallis test followed by the Dunn’s post-test were used. Statistical significance was determined and recorded as follows: *p* < 0.001 (***), *p* < 0.01 (**), *p* < 0.05 (*), or not statistically significant if *p* > 0.05.

## 3. Results

### 3.1. Isolation and General Characteristics of Enterococcus Phage A2

*Enterococcus* phage vB_EfaM_A2 was isolated from farm animal faeces obtained in Co. Cork, Ireland, in September 2017. The bacterial host used for its isolation was *E. faecalis* strain OG1RF (rifampicin and fusidic acid resistant derivative of strain OG1), a human isolate that has been described to induce intestinal inflammation in mouse models of experimental colitis [13,50]. On TSB agarose overlays (0.2% *w*/*v*) phage A2 forms clear plaques on OG1RF with diameters ranging between 0.5 mm and 1.0 mm (Supplementary information 1, Figure S2). The phage possesses a relatively wide host range, infecting 13 of 20 tested strains of *E. faecalis* (data not shown).

**Figure 1:**
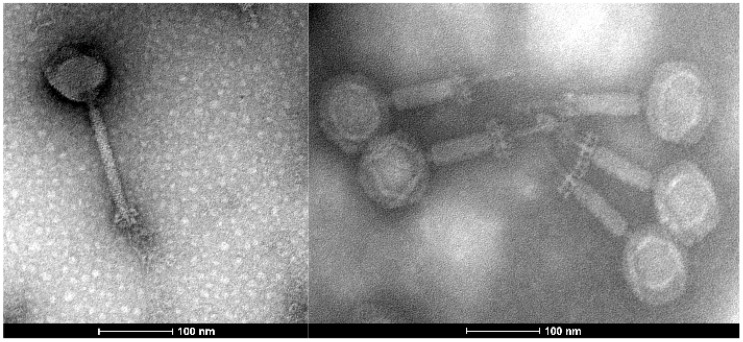
Electron micrograph of *Enterococcus* phage A2. Phage particles were negatively stained with 0.5% (*w*/*v*) uranyl acetate. Scale bar represents 100 nm.

Transmission electron microscopy revealed that phage A2 has an A1 morphotype [51], with an icosahedral head of 83.39 nm × 83.40 nm (*n* = 5) and a contractile tail of 173.07 nm × 18.64 nm (*n* = 5) (Figure 1). When its tail is uncontracted, the virion possesses a similar morphology and a “clumped/aggregated” base plate appendage as described for phages of *Kayvirus* [24]. The phage was named in accordance with the nomenclature set out by Kropinski et al. [52].

### 3.2. Genome Sequence of Enterococcus Phage A2

Phage A2 has a genome of 149,431 bp with a G + C content of 37%, similar to its host *E. faecalis* OG1RF GC content of 37.8% [53]. The A2 genome has 191 open reading frames (ORFs) with gene products ranging in size from 30 to 2198 amino acids. Eighty-eight ORFs (46%) could be annotated (Supplementary information 2, Table S1) with others being identified as putative membrane proteins (10), putative lipoproteins (2), and hypothetical proteins (91). A search of these proteins against the Prokaryotic Virus Orthologous Groups (pVOGs) database could assign 132 to orthologous groups. Additionally, the genome possesses 24 tRNA genes encoding for 19 amino acids.

**Figure 2:**
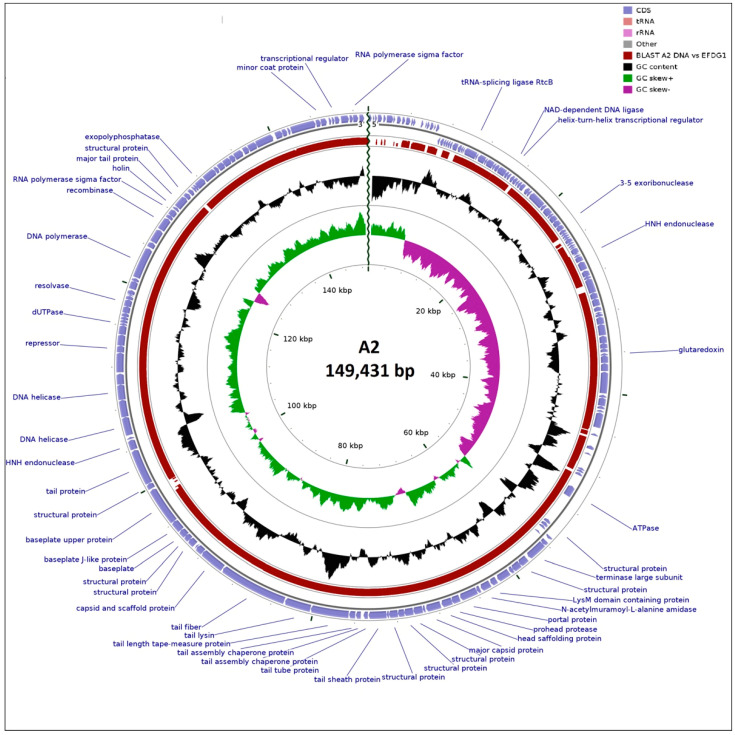
Summary of the genomic organization of *Enterococcus* phage A2. The outer ring shows putative ORFs, represented by purple arrowheads labelled with a predicted function where possible. The following inner ring shows a TBLASTX comparison (in red, where homologous) to *Enterococcus* phage EFDG1. GC content is depicted in black, while positive and negative GC skew is denoted by green and purple, respectively.

When the genome is viewed as a single circular contig, the orientation of ORFs divides it into two main gene clusters, with GC skew strongly correlating with this observation (Figure 2). Region 1 spans 81 ORFs from A2_19 to A2_99, with region 2 consisting of 110 ORFs spanning A2_100 to A2_18. Annotation obtained from BLASTP (nr database), hmmscan (Pfam and pVOG databases), and HHpred analysis (PDB and Pfam databases) of the ORFs in cluster 1 indicated that they are mostly associated with roles in metabolism. Cluster 2 ORFs are linked with roles involved in DNA replication, virion structure, and peptidoglycan degradation. Screening against the CARD database [54] indicates that the genome encodes no gene products associated with antibiotic resistance. Furthermore, the phage likely follows an exclusively lytic lifestyle as no integrase, excisionase, or repressor proteins were identified. PHACTS also predicts the phage to be lytic (0.511 ± 0.075).

### 3.3. Ig-Like Domains Associated with Enterococcus Phage A2 and the *Herelleviridae* Family

Phage virion structural proteins with Ig-like domains have been implicated in the weak binding interactions that can occur between phage and the mucin-displayed glycan associated with mucosal surfaces, in keeping with the bacterial adherence to mucus (BAM) model [55]. For phages with contractile tails, proteins which form the phage virion that have been found to possess Ig-like domains include the Hoc protein, fibritin, the major tail, and proteins that form the baseplate. The Ig-like domains that occur among phages can be placed into three distant sequence families: bacterial Ig-like domain 2 (Big 2), immuno-globulin superfamily (I-Set), and fibronectin type III domain (FN3) [56].

Twenty-nine ORFs were annotated as having a role in the formation of the phage A2 virion, such as DNA packaging, assembly chaperones, scaffolding, and structure. Of the structural proteins associated with the formation of the capsid, tail, baseplate, and tail fibres of the phage, two ORFs possess the bacterial Ig-like group 2 domain (IPR003343, PF02368.18). These two ORFs, A2_133 and A2_164, were annotated as a putative baseplate protein and major tail protein, respectively. The structural role of these proteins in the virion of A2 is supported by homology of A2_133 to gp104 (accession no. AAY52885.1, E-value = 8 × 10^−88^) of *Listeria* phage A511, and homology of A2_164 with gp70 (accession no. CCA65802, E-value = 5 × 10^−25^) and gp71 (accession no. CCA65802, E-value = 6 × 10^−17^) of *Staphylococcus* phage ISP. These proteins have been confirmed by mass spectrometry to be part of the virion of these phages [23,57].

**Figure 3:**
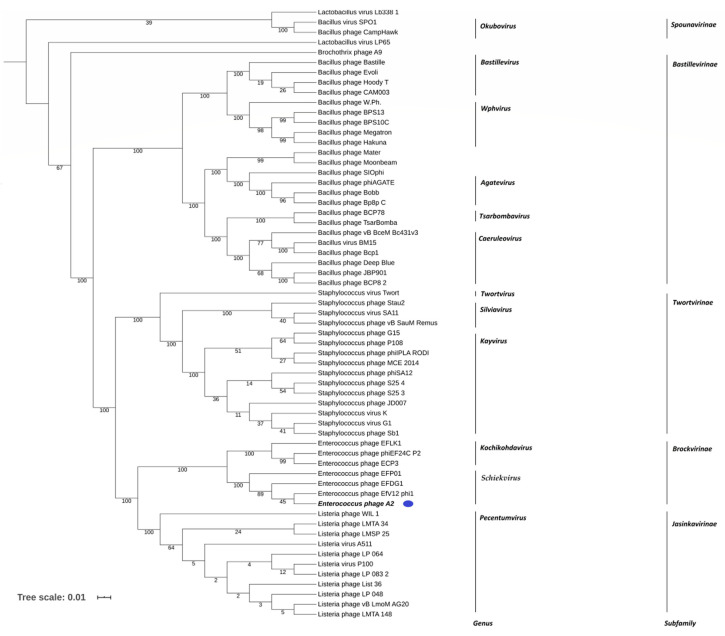
Amino acid based VICTOR-generated phylogenomic Genome-BLAST Distance Phylogeny (GBDP) tree of *Enterococcus* phages A2 (highlighted with a blue dot), EfV12-phi, and EFP01 with 58 members of the *Herelleviridea* family Inferred using the formula D4 and yielding average support of 69%. The numbers below branches are GBDP pseudo-bootstrap support values from 100 replications.

At the DNA level, the closest relatives of phage A2 are *Enterococcus* phages EFDG1 (accession no. NC_029009.1), EfV12-phi (accession no. MH880817.1), and EFP01 (accession no. KY549443.1), all of which share high genome synteny (Supplementary information 1, Figure S3) with >70% nucleotide identity (BLASTN) and shared proteome content of >80% (Coregenes 3.5) (Supplementary information 2, Table S2). Moreover, analysis of phage A2 shows that it possesses significant homology at the protein level to phages belonging to the recently established *Herelleviridae* phage family. This family was formed to contain what was previously classified as the *Spounavirinae* subfamily (*Bacillus* phage SPO1-related viruses), with detailed comparative analysis showing them to be clearly distant from other members of the family *Myoviridae* [58]. Whole-genome comparison based on amino acid sequences was performed using VICTOR shows that they belong to the genus *Schiekvirus* within the family of *Herelleviridae* (Figure 3). Additionally, analysis using Gegenees (TBLASTX), based on protein similarity, also indicated that the A2-like phages belong to this genus sharing identity between 80% and 90% and only possessing an identity of only 41–45% with phages of the genus *Kochikohdavirus* (Supplementary information 1, Figure S4).

To better understand the distribution of the Ig-like domain containing proteins associated with phage A2 and the 58 phages currently recognized by the ICTV as belonging to the family of *Herelleviridae*, their proteomes were analyzed with the Pfam database. A total of 282 pFam domains could be identified with 24 of the 59 phages found to possess the bacterial Ig-like domain (Big 2, PF02368.13) (Supplementary information 2, Table S3). Only phages infecting the bacterial genera of *Listeria*, *Enterococcus, Staphylococcus,* and *Lactobacillus* were found to possess this domain, and it was not found among phages infecting *Bacillus*. *Enterococcus* phage A2 is the only phage possessing two proteins with the Big 2 domain, all other phages have only a single protein with the domain. These proteins range between 123 and 305 amino acids in length with many having been annotated as a major tail proteins on GenBank (Supplementary information 1, Figure S5 and Supplementary information S2, Table S4). Additionally, one other class of Ig-like domain from the FN3 family (PF00041.16) was found to occur on the proteome of *Bacillus* phage SIOphi (accession no. KC699836).

### 3.4. Phage Infection and E. faecalis Growth in Cell Culture Media

**Figure 4:**
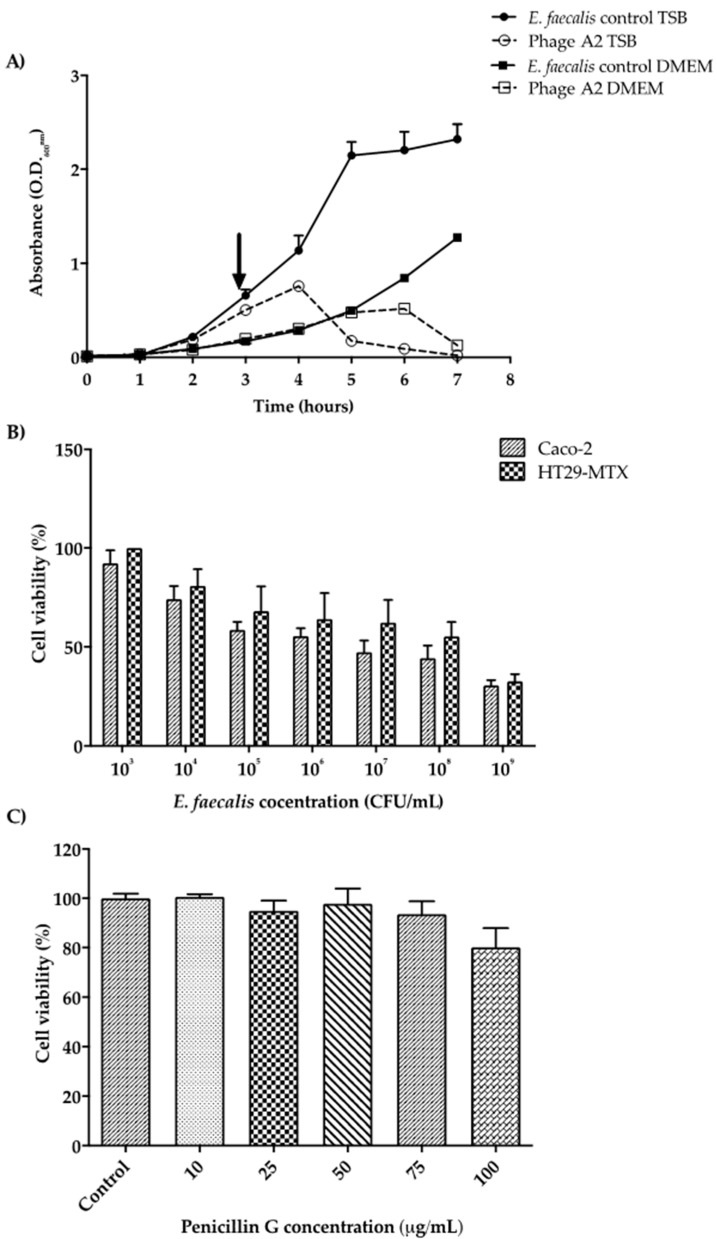
(**A**) *Enterococcus* phage A2 infectivity and *E. faecalis* growth in TSB broth and DMEM cell culture media. Results show average values (*n* = 3) ± standard error (arrow indicates timepoint which phage A2 was introduced to culture). (**B**) Caco-2 and HT29-MTX cell viability following infection of *E. faecalis* at varying concentrations. The cells were exposed to *E. faecalis* for 24 h before measuring survival rates. Columns show average cell viability in percentage (*n* = 6) ± standard error. (**C**) Caco-2 and HT29-MTX co-culture viability at varying penicillin G concentrations. Columns show average cell viability in percentage (*n* = 3) ± standard error.

Before studying the interaction between phage A2 and its host in a mucus barrier model, we determined their propagation in DMEM cell culture media used to propagate and maintain the cell lines. As a control, the same experiment was carried out using the TSB used as the standard growth media for *E. faecalis* throughout this study. *E. faecalis* grew more slowly in DMEM, with an extended lag phase and had not reached stationary phase after 7 h of incubation (Figure 4A). Phage A2 also killed the host faster in TSB broth, causing a drop in O.D_600nm_ 1 h after infection. Killing was not observed in DMEM until 3 h post-infection. However, a similar level of lysis was achieved 7 h post-infection (Figure 4A).

### 3.5. Caco-2 and HT29-MTX-E12 Cell Viability Following E. faecalis Infection and Treatment with Penicillin G

Caco-2 cells and HT29-MTX cells were individually infected with varying levels of *E. faecalis*, from 10^3^ to 10^9^ CFU/mL (Figure 4B). As expected, for both cell lines there was a gradual reduction of viability with increasing numbers of *E. faecalis*. Cell viability was not affected with the presence of *E. faecalis* at the lowest concentration (10^3^ CFU/mL) for both cell lines when compared to the controls (100 ± 0.0% for HT29-MTX and 92.35 ± 3.4% for Caco-2 cells). However, the exposure to 10^9^ CFU/mL of *E. faecalis* increased cell mortality with survival levels of only 32.6 ± 3.6% for HT29-MTX and 30.5 ± 2.7% for Caco-2 cells after 24 h exposure. *E. faecalis* at 10^6^ CFU/mL resulted in ~55% viability of both cell lines (Figure 4B). This level was selected to perform further experiments with a co-culture of Caco-2/HT29-MTX cells. We also investigated the toxicity of penicillin G (10 to 100 µg/mL) against the co-culture. Twenty percent cell mortality was observed for the highest concentration of the antibiotic (100 μg/mL) (Figure 4C). A concentration of 75 μg/mL had no impact on cell viability but was lethal for *E. faecalis* after 2 h exposure (data not shown). Therefore, this concentration was selected for the invasion experiments.

### 3.6. Interaction between Adhered E. faecalis and Phage A2 in Intestinal Epithelium with and without Mucus

**Figure 5:**
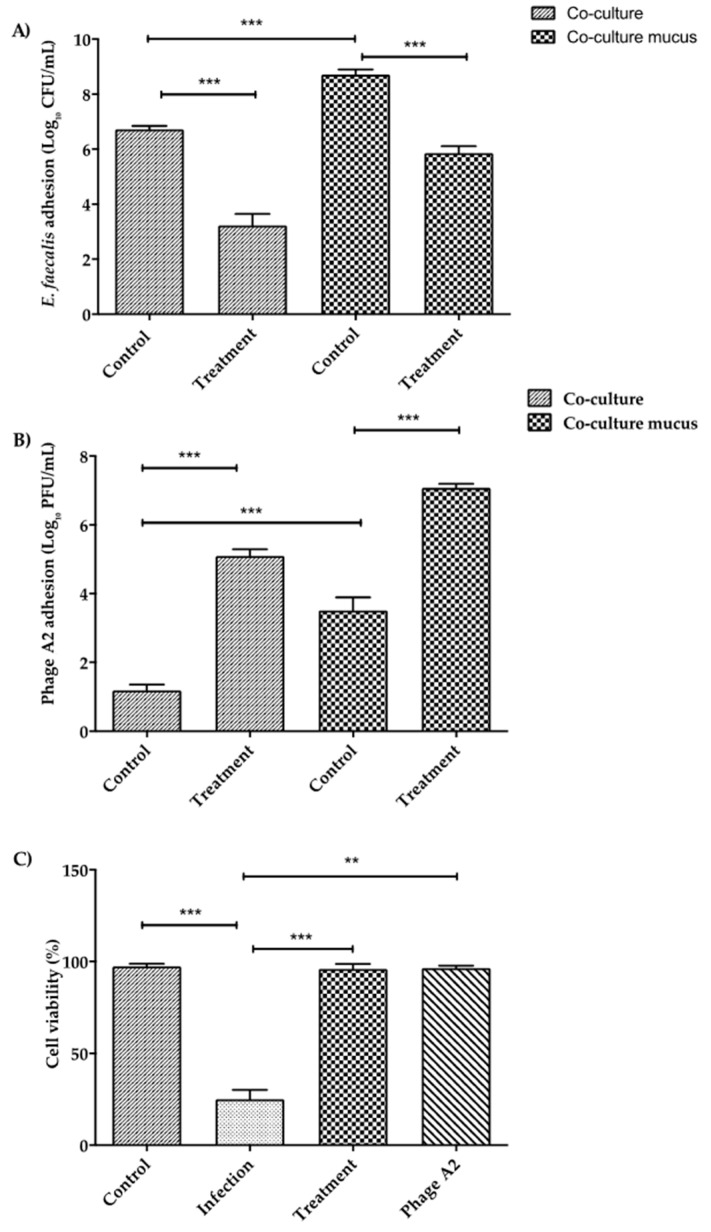
(**A**) *Enterococcus* phage A2 and adhered *E. faecalis* interaction in a co-culture mimicking the intestinal epithelium with and without mucus. *E. faecalis* was left to adhere to the co-cultures for 3h. At this point, fresh media was added and the treatment (*E. faecalis* + phage A2) groups were inoculated with phage A2. (**B**) *Enterococcus* phage A2 adherence to a co-culture with and without the presence of mucus. Control groups were co-cultures only inoculated with phage. The treatment groups were infected with *E. faecalis* for 3 h and then treated with phage. All the samples were collected at 24 h post-inoculation. (**C**) Cell survival of co-culture producing mucus following 24 h. of exposure to *E. faecalis* and *Enterococcus* phage A2. Cell survival following each exposure is shown in comparison to a control co-culture without bacteria and phage. All values are average concentrations (*n* = 9) ± standard error. ** *p* < 0.01; *** *p* < 0.001.

In order to understand the behavior of phage A2 when infecting *E. faecalis* in the presence of intestinal epithelial cells, Caco-2/HT29-MTX co-cultures were infected with 10^6^ CFU/mL *E. faecalis* for 3 h before removing planktonic bacteria. Then, cells were washed with DPBS and phage A2 was added at a concentration of 10^6^ PFU/mL for 21 h to assess its impact on bacterial populations that had adhered to the cell surface. The presence of mucus significantly enhanced the ability of *E. faecalis* to adhere to the epithelium surface (8.71 ± 0.18 log) when compared to co-culture without mucus (6.71 ± 0.35 log, Figure 5A, *p* < 0.001). The addition of phage reduced the levels of *E. faecalis* attached to the cell surface and mucus in both cases in approximately 3-log, with an average of 3.22 ± 0.41 log and 5.85 ± 0.25 log, respectively (Figure 5A, *p* < 0.001).

Phage A2 at a concentration of 10^6^ PFU/mL added to the Caco-2/HT29-MTX co-culture without the host could only be detected at very low levels, with an average of 1.1 ± 0.17 log after 24 h adhesion. However, in the same situation with cells producing mucus, phage A2 was present at significantly higher levels of 3.5 ± 0.38 log (Figure 5B, *p* < 0.001).

### 3.7. Epithelial Cells Co-Culture Response during Phage A2 and E. faecalis Interaction

We next examined the impact of phage and bacteria on the viability of the epithelial cells in Caco-2/HT29-MTX co-culture producing mucus. As expected, the addition of 10^6^ CFU/mL of *E. faecalis* caused a major reduction in cell survival, with only 25 ± 5.1% survival after 24 h (Figure 5C, *p* < 0.001). The presence of phage A2 on its own did not induce any cell mortality, despite the presence of bacterial host debris in the filtered sterilized phage lysate (Figure 5C), which coincides with the results observed in co-cultures without mucus (data not shown). The Caco-2/HT29-MTX co-culture infected with *E. faecalis* and subsequently inoculated with phage A2 had the same viability (96.0 ± 2.7) as the control uninfected group (97.3 ± 1.4) (Figure 5C, *p* < 0.001). This indicates that phage A2 can target *E. faecalis* and protect the epithelial cells from bacterial induced cell death.

### 3.8. E. faecalis and Phage A2 Translocation through the Intestinal Barrier

**Figure 6:**
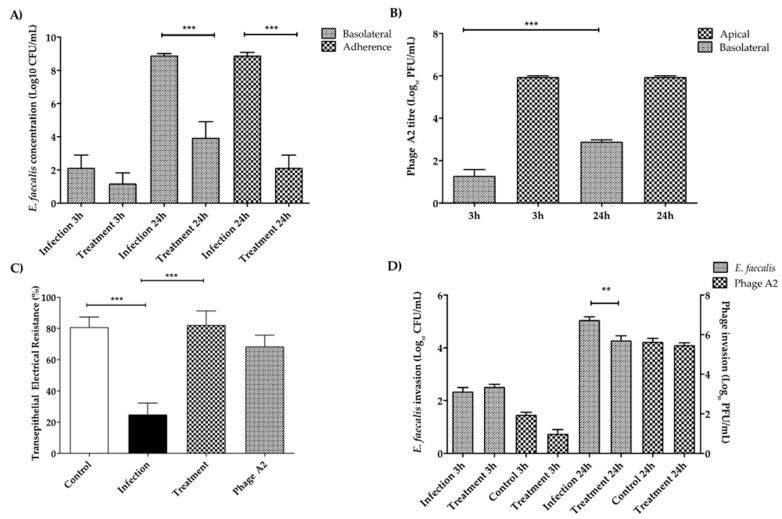
(**A**) *E. faecalis* adherence and translocation through intestinal epithelium and the effect of phage A2 predation. *E. faecalis* was left to adhere to the co-cultures for 3 h. At that point, treatment groups were inoculated with phage A2. (**B**) *Enterococcus* phage A2 translocation through intestinal epithelium when grown in trans-wells. Phage A2 was left to adhere to the co-cultures for 3 h. Then, fresh media was added to the wells without *E. faecalis*. (**C**) Co-culture trans-epithelial electrical resistance during exposure to *E. faecalis* and *Enterococcus* phage A2. *E. faecalis* was left to adhere to the co-cultures for 3 h. Treatment groups were inoculated with phage A2. The control and the phage groups were left uninfected with *E. faecalis*. (**D**) *E. faecalis* and phage A2 invasion of the intestinal epithelium. *E. faecalis* was left to adhere to the co-cultures for 3 h. At that point, the treatment groups were inoculated with phage A2. After 3 h or 24 h the samples were treated for 2 h with penicillin G to remove all the extracellular *E. faecalis*. Phage controls were not infected with *E. faecalis*, these groups were used to check phage invasion without the host. Columns show average concentration (*n* = 9) ± standard error. ** *p* < 0.01; *** *p* < 0.001.

We also investigated the ability of phage to cross the intestinal barrier. Caco-2/HT29-MTX co-cultures were allowed to form a layer and produce mucus in trans-wells. The co-cultures were infected with *E. faecalis* at a concentration of 10^6^ CFU/mL and treated with 10^6^ PFU/mL of phage. After 3 h, *E. faecalis* had crossed the epithelial cell barrier at a very low level (log 2.1 ± 0.8), and the presence of phage had no impact. However, after 24 h the number of *E. faecalis* found in the basolateral compartment was much higher, at log 8.9 ± 0.1. In this setting, the presence of the phage A2 significantly impacted the translocation of *E. faecalis*, reducing its numbers by up to 5-log (Figure 6A, *p* < 0.001). This pattern is reflected in the adherence of *E. faecalis* to the mucus layer, with higher concentrations found in the untreated infected cultures (8.9 ± 0.2) and reduced numbers in treated cultures (Figure 6A, *p* < 0.001).

Phage A2 adhered to the mucus layer (3.3 ± 0.3 log) at levels that were very similar to those found in the mucus producing co-cultures grown in 96 well plates (Figure 5B). The phage was also able to translocate to the basolateral compartment in the absence of its host bacterium (Figure 6B). The translocation increased with time from 1.8 ± 0.3 log at 3 h to 2.9 ± 0.1 log after 24 h (Figure 6B, *p* < 0.001). The phage found in the basolateral compartment represented 0.4% of the total phage found on the apical side.

The integrity of the epithelium was determined during the infection by measuring trans-epithelial electrical resistance (TEER) (Figure 6C). As expected, the presence of *E. faecalis* severely compromised the integrity of the cell layer, reducing the TEER to 25.0 ± 7.2% (Figure 6B, *p* < 0.001). In the presence of phage A2 the structure of the epithelium was preserved, with a TEER value close to the uninfected control cultures (82.3 ± 8.9%; Figure 6D, *p* < 0.001).

### 3.9. E. faecalis and Phage A2 Invasion in Intestinal Cell Line Co-Cultures

After 24 h of the addition of *E. faecalis* at 10^6^ CFU/mL, *E. faecalis* was found to have invaded the epithelial cells (5.1 ± 0.1 log, Figure 6D), almost 4-log fewer than those that had translocated through the layer (Figure 6B). The number of internalized *E. faecalis* was reduced by 1-log in the presence of phage A2 (10^6^ PFU/mL initial concentration) (Figure 6D, *p* < 0.01). However, the number of phage entering the cells was the same in the treatment and the uninfected phage control group, indicating that phage did not replicate inside the epithelial cells (Figure 6D).

## 4. Discussion

Phages have been used therapeutically since 1917 [59]. However, most phage characterization examines the relationship with the target bacteria without considering a eukaryotic host. In the presence of a eukaryotic host, the performance and behavior of the phage will likely vary significantly. Here we describe the interaction of phage A2 and its host bacterium *E. faecalis* with respect to a mucus producing intestinal epithelial layer, better mimicking the conditions found in the mucosal layer of the human gut [49]. In this model, we could judge the potential therapeutic value of the phage as well as understand the translocation, adhesion, and invasion of both phage and target bacteria. We were also able to examine the viability of the eukaryotic cells and the integrity of the epithelial barrier in the presence and absence of phage. A number of recent studies have used in vitro cell culture models to test the behavior of phage in the gut and their activity against intestinal pathogens [60,61]. To date, most have used single cell monolayers without a mucus layer [60,61]. More complex models using co-cultures have been utilized for the interaction of the phage with the epithelial layer. However, these studies are limited by the absence of a host bacteria, with none considering the impact that a cell culture medium can have on the fitness of both phage and bacteria [62,63]. In the case of *E. faecalis* and phage A2, there was a noticeable difference in the growth and infectivity patterns observed in TSB broth or DMEM glutamax (Figure 4A). A delay in the growth of *E. faecalis* was evident which was linked with prolonged and slower lysis by phage A2 (Figure 4B). These factors reveal how environmental changes can have a significant effect on the interaction between the test phage and host bacteria and should be taken into account during the experimental design and selection of phages for therapy.

The presence of immunoglobulin (Ig) domains in phage virion proteins have been associated with their ability to adhere to mucus and display increased predatory ability in the gut [55]. Several phages from the family *Herelleviridae* have been shown to have therapeutic promise and are constituents of commercially available phage cocktails [24]. The presence of Ig-like domains on their virions may play an important role in their therapeutic value. Comparative analysis in this study shows that only phages of *Herelleviridae* infecting *Listeria*, *Enterococcus*, *Staphylococcus,* and *Lactobacillus* possess proteins with an Ig-like domain of the Big 2 family (PF02368.13). Apart from *Bacillus* phage SIOphi possessing the Ig-like domain of the FN3 family (PF00041.16), no other *Herelleviridae* phage infecting *Bacillus* was identified to possess a proteome with Big 2 (Supplementary information 1, Figure S5 and Supplementary information 2, Table S4). It is tempting to speculate if this domain provides an advantage to phages of *Herelleviridae* infecting bacteria that can easily be associated with a mucosal environment. *Enterococcus* phage A2 belongs to the genus of *Schiekvirus* of the family *Herelleviridae*. The phage is an uncommon member of this family, possessing a Big 2 domain in both a putative major tail protein and also in a protein believed to form part of its baseplate.

The presence of mucus increased the adherence of the phage A2 to the cell layer (Figure 5B, *p* < 0.001); this property may be related to the Ig-like domains associated with its virion. However, further investigation will be required to verify such a hypothesis. The presence of mucus also increased the adhesion of *E. faecalis,* highlights the importance of including a mucus layer as it has an influence on the interaction between the phage, the host bacteria and the epithelium.

The integrity of cellular barriers acts as a marker for the health of the intestine as these barriers regulate diffusion and transport across cellular membranes. Intestinal barrier dysfunction has been associated with Crohn’s disease [64]. Certain *E. faecalis* strains encode cytolysins [17] and gelatinases [11,12], these enzymes can weaken cellular junctions causing increased bacterial translocation through the gut barrier leading to inflammation and disease. Recent reports showed that phage therapy against such intestinal strains attenuates alcoholic liver disease [17]. Our epithelial co-culture model reveals a correlation between the presence of *E. faecalis* and reduced integrity of the epithelial barrier, significantly reduced TEER values and higher translocation of the bacteria. The presence of phage A2 controlled the population of *E. faecalis* and preserved the integrity of the tight junctions (Figure 6C, *p* < 0.001) and clearly reduced pathogen adhesion and translocation (Figure 6A, *p* < 0.001), in agreement with previously successful phage therapy against *E. faecalis* [17]. Another finding was that phage A2 could translocate across the epithelial barrier in our model (Figure 6B). This finding gives additional weight to the ever interesting awareness that phage can cross eukaryotic cell barriers giving explanation to their detection in tissues and organs of the human body [65,66,67]. In summary, our model indicates the *Enterococcus* phage A2 (a member of *Herelleviridae*) has a potential therapeutic value against *E. feacalis* infection in an intestinal epithelium-like environment.

In this study, we developed a simple in vitro cell culture model to test the interaction between phage and host bacteria in an environment that better represents the intestinal epithelium. This model allows the characterization of bacterial pathogenicity and features of phage that can influence their performance in vivo, including aspects such as adherence to mucus and translocation. Simultaneously, both cell viability and tight junction integrity can be assessed. We believe this system represents a cost effective platform suitable to support the selection of potentially therapeutic phages.

## Supplementary Materials

The following are available online at https://www.mdpi.com/2076-2607/8/9/1374/s1, Figure S1: Experimental design of Caco-2/HT29-MTX co-culture experiments. Figure S2: TSB agarose (0.2% *w*/*v*) overlay with *Enterococcus faecalis* strain OG1RF and *Enterococcus* phage A2. Figure S3: Comparison of the genome of *Enterococcus* phage A2 with closest relatives. Figure S4: Genenees TBLASTX heatmap analysis of the phages which constitute the family *Herelleviridae*. Figure S5: Ig-like domains found among phages of *Herelleviridae.* Table S1: Genome annotation of *Enterococcus* phage A2. Table S2: GenBank details of phages belonging related to *Enterococcus* phage A2. Table S3: Complete list of pFam domains identified in the set of 59 Herellevirus genomes. Table S4: Details of phages of *Herelleviridae* and Ig-like domains associated with them.
