# Supplementary material for: Characterizing Phage-Host Interactions in a Simplified Human Intestinal Barrier Model
Source: Microorganisms. 2020 Sep 7;8(9):1374. doi: 10.3390/microorganisms8091374 (PMC7563437; doi:10.3390/microorganisms8091374)
Supplement: Supplementary file 1 [file microorganisms-08-01374-s001.zip › Supplementary material/Supplementary information 1.docx]

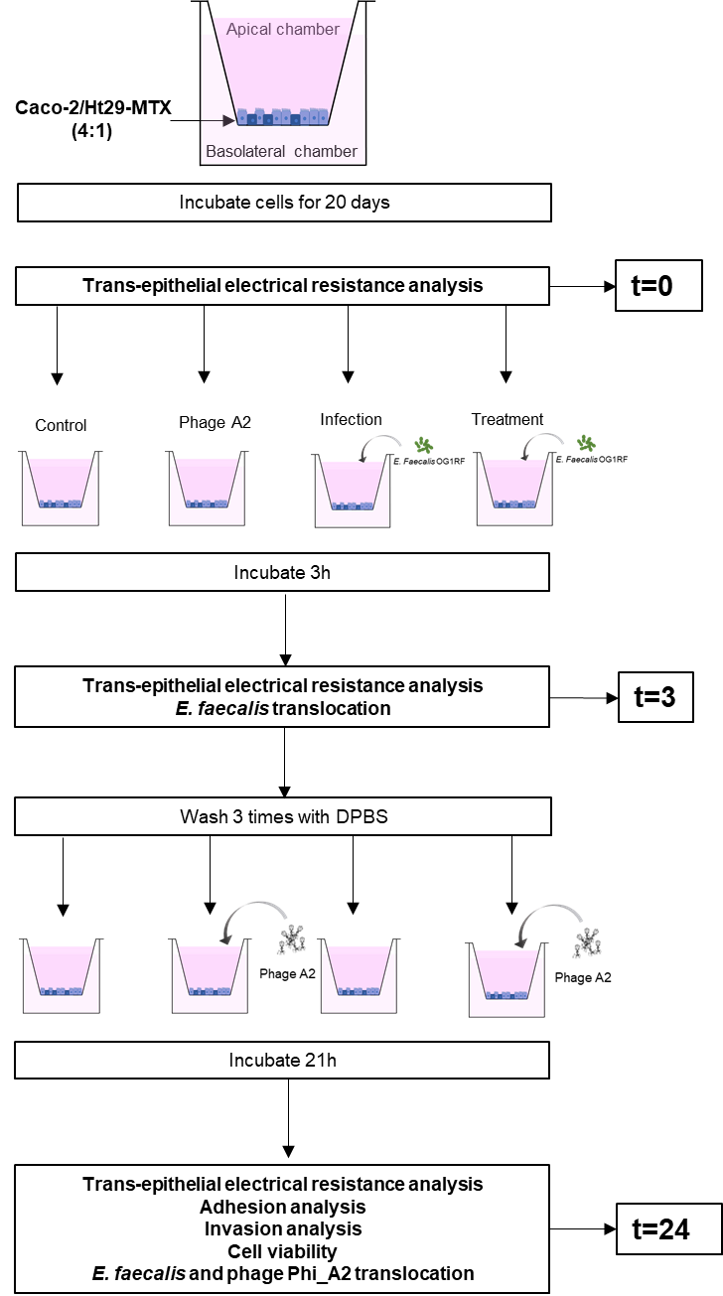


**Figure S1.** Experimental design of long-term Caco-2/HT29-MTX co-culture experiments. Cells were incubated for 20 days to allow formation of mucus layer. At t=0 trans-epithelial electrical resistance (TEER) was determine before starting the experiments. *E. faecalis* OG1RF at a concentration of 10^6^CFU/mL was added to the wells belonging to *E. faecalis* group and treatment group. The remaining groups (control and phage A2) were incubated with sterile growth media. After 3 h of incubation TEER values were determined for all wells. To determine *E. faecalis* translocation 30 μL of media from the apical and the basolateral chambers were removed and plated in TSB agar. Then, cells were washed 3 times with Dulbecco’s Phosphate Buffered Saline (DPBS) in order to remove any remaining bacteria. Fresh media was added to both apical and basolateral chambers and phage A2 was added to the pertinent groups (Phage A2 and treatment groups). Cells were incubated for 21 h prior to analyse TEER, adhesion, invasion, cell viability and *E. faecalis* and phage A2 translocation.


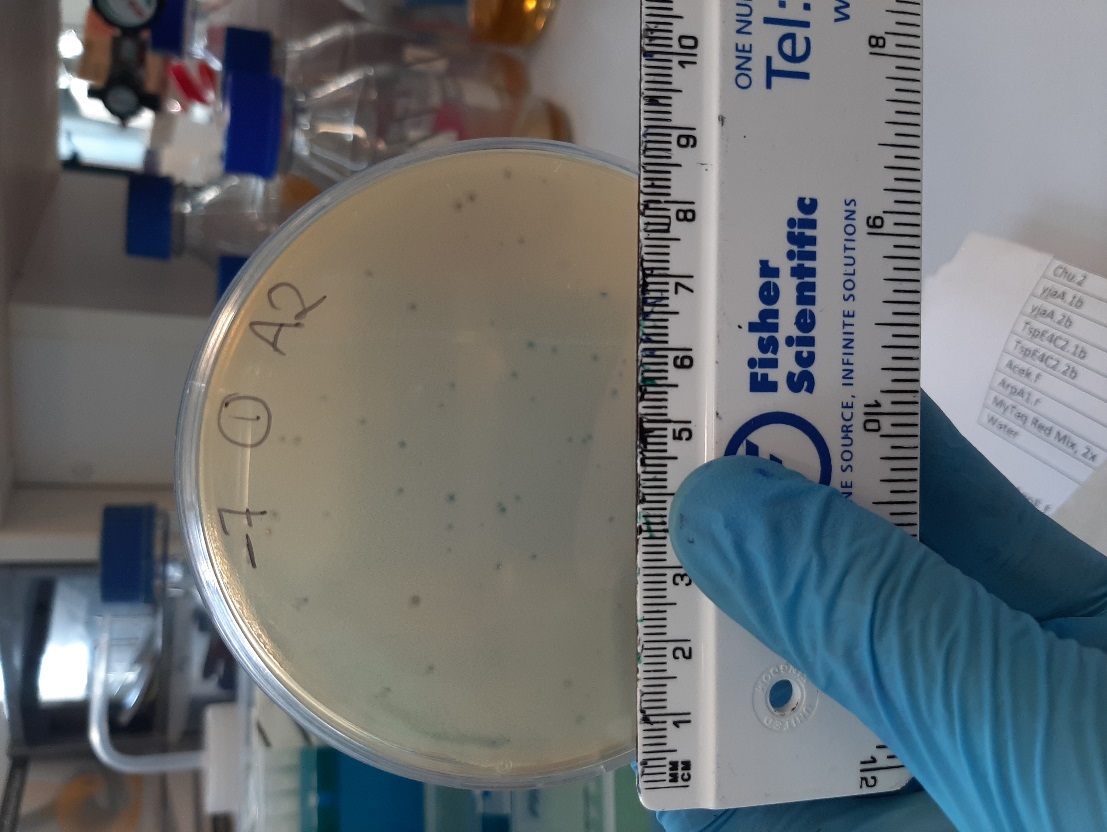


**Figure S2.** TSB agarose (0.2% w/v) overlay with *Enterococcus faecalis* strain OG1RF and *Enterococcus* phage A2.


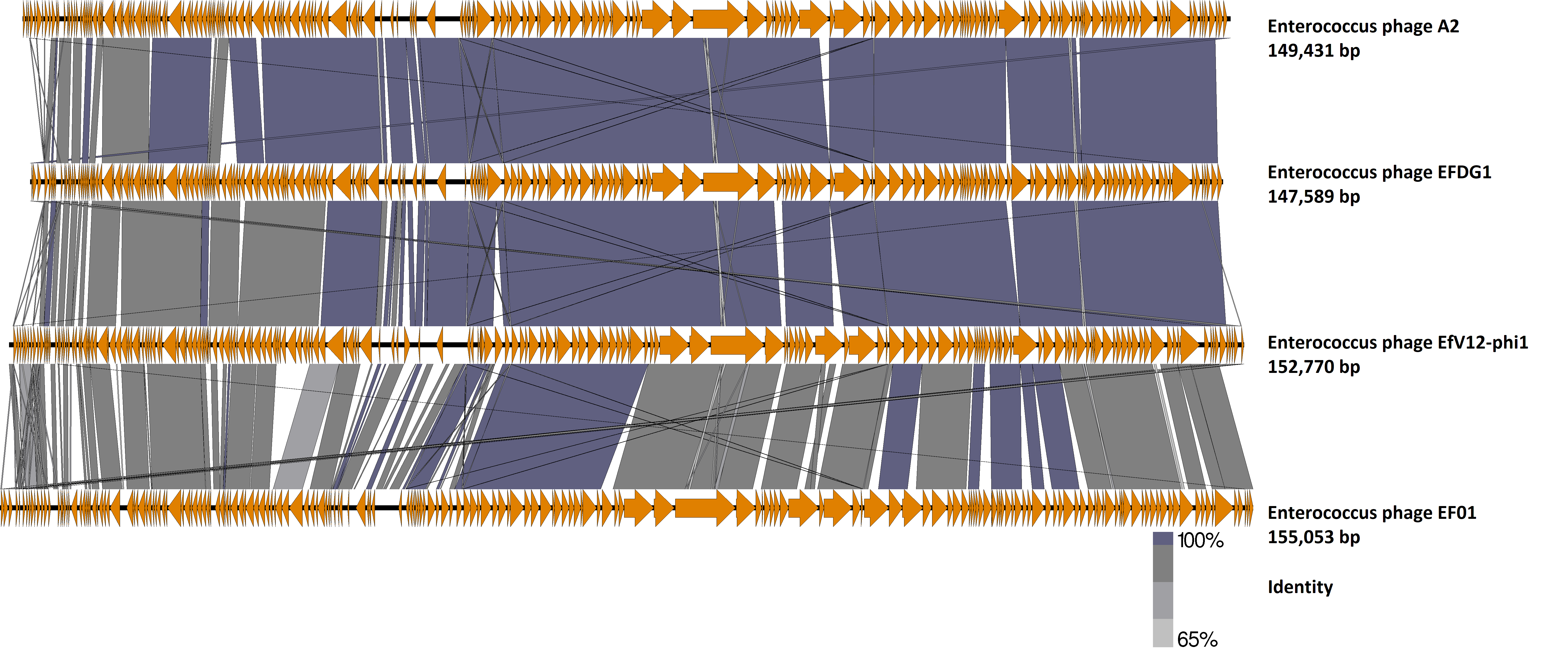


**Figure S3.** Comparison of the genome of *Enterococcus* phage A2 with closest relatives using currently available annotations employing BLASTN and visualised with Easyfig.

**Figure S4.** Gegenees TBLASTX heat map analysis of the phages, which constitute the family *Herelleviridae*. *Enterococcus* phage A2 is highlighted in blue. Using accurate parameters, fragment length: 200 bp; and step size: 100 bp with the threshold set to 0%


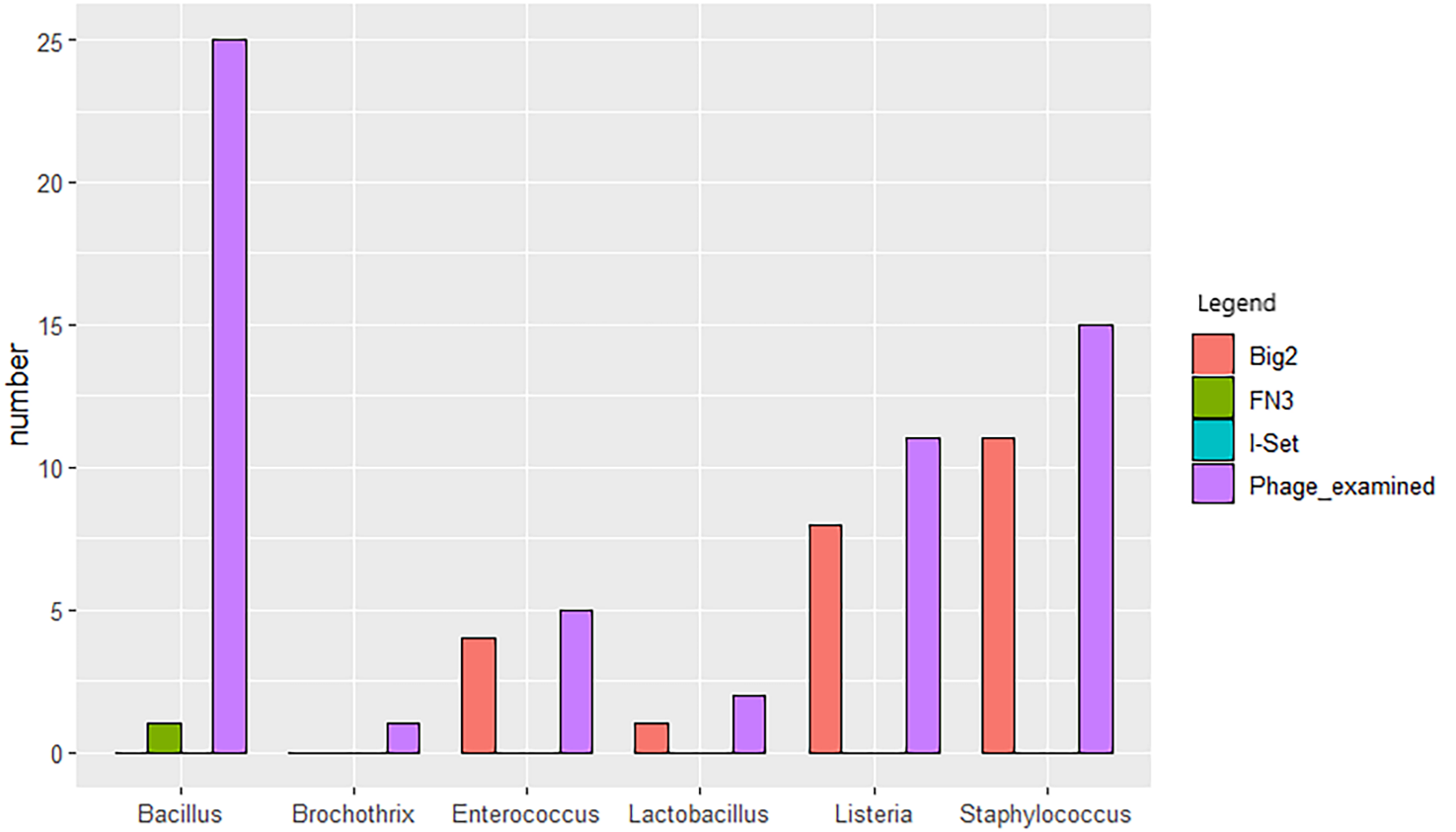


**Figure S5.** Ig-like domains found among phages of *Herelleviridae.* The number of phages of *Herelleviridae* infecting particular bacteria genera examined for Ig-like domains types (purple) found to possess either Big2 (red), FN3 (green) or I-Set (blue) domains.

**Table S2**. Genbank details of phages related to *Enterococcus* phage A2

| phage | Accession | DNA sequence identity (%)* | Shared proteins (%)  ** | GC% | Genome length (bp) | ORFs | tRNA*** |
| --- | --- | --- | --- | --- | --- | --- | --- |
| Enterococcus phage A2 | MT856905 | 93 | 89.06 | 37 | 149,431 | 191 | 24 |
| Enterococcus phage EFDG1 | NC_029009.1 | 100 | 100 | 37 | 147,589 | 192 | 24 |
| Enterococcus phage EfV12-phi1 | MH880817.1 | 93 | 88.02 | 37 | 152,770 | 191 | 24 |
| Enterococcus phage EFP01 | KY549443.1 | 81 | 79.17 | 37 | 155,053 | 193 | 7 |

* BLASTN (against EFDG1), **Coregenes 3.5 (against EFDG1), *** determined with ARAGORN
